# Lab-on-Fiber Sensors with Ag/Au Nanocap Arrays Based on the Two Deposits of Polystyrene Nanospheres

**DOI:** 10.3390/polym15204107

**Published:** 2023-10-16

**Authors:** Meng Shi, Shifang Gao, Liang Shang, Linan Ma, Wei Wang, Guangqiang Liu, Zongbao Li

**Affiliations:** 1 School of Physical Science and Intelligent Engineering, Jining University, Qufu 273155, China; 2 Shandong Provincial Key Laboratory of Laser Polarization Technology, Qufu Normal University, Qufu 273165, China; 3 Ministry of Education Key Laboratory of Textile Fiber Products, School of Materials Science and Engineering, Wuhan Textile University, Wuhan 430200, China; 4 School of Materials and Chemical Engineering, Tongren University, Tongren 554300, China

**Keywords:** lab-on-fiber sensor, Ag/Au nanocap arrays, surface-enhanced Raman scattering, polystyrene nanospheres

## Abstract

Surface-enhanced Raman spectroscopy (SERS) can boost the pristine Raman signal significantly which could be exploited for producing innovative sensing devices with advanced properties. However, the inherent complexity of SERS systems restricts their further applications in rapid detection, especially in situ detection in narrow areas. Here, we construct an efficient and flexible SERS-based Lab-on-Fiber (LOF) sensor by integrating Ag/Au nanocap arrays obtained by Ag/Au coating polystyrene nanospheres on the optical fiber face. We obtain rich “hot spots” at the nanogaps between neighboring nanocaps, and further achieve SERS performance with the assistance of laser-induced thermophoresis on the metal film that can achieve efficiency aggregation of detected molecules. We achieve a high Raman enhancement with a low detection limitation of 10^−7^ mol/L for the most efficient samples based on the above sensor. This sensor also exhibits good repeatability and stability under multiple detections, revealing the potential application for in situ detection based on the reflexivity of the optical fiber.

## 1. Introduction

Surface-enhanced Raman spectroscopy (SERS) is considered as one of the most sensitive techniques for non-destructive, single-molecular detection, with significant advantages of characterizing the fingerprint spectrum and having a short detection time [1,2,3,4,5]. Despite the mechanism of the chemical and electromagnetic interaction occurring between an analyte and metal nanostructure surface in SERS detection, the surface area available for analyte detection plays an important role for SERS. This is also one purpose of the conventional SERS substrates constructed with micro/nanostructures [6,7,8]. To date, great efforts have been made to modulate the metal micro/nanostructure on rigid, flat-based substrate to achieve high surface area availability. However, this kind of SERS sensor lacks integration capabilities, limiting its further applications such as in situ detection in in vivo environments.

Lab-on-Fiber (LOF) offers avenues for continued increases in SERS detection based on the advantages of high portable abilities, high biocompatibility, and high sensitivity [9,10]. The LOF device can further integrate nanostructure and functional materials on the fiber tip to achieve specific purposes, and is widely used in material analysis [4], biomedical engineering [11], biosensor application [12], and explosive detection [13]. Conventional LOF devices require precise fabrication employing complex nanotechnology, causing high device costs, and limiting their application. Specifically, superior to conventional SERS substrates supported by silicon wafer or quartz glass with smooth and flat surfaces, PS nanospheres can provide a hexagonal close-packed, three-dimensional (3D) structure with eminently ordered V-shaped nanogaps, yielding a high density of electromagnetic “hot spots” when noble metal materials are decorated [14]. Developing a low cost but highly efficient SERS-based LOF sensor requires us to find another way. 

Thermophoresis provides an efficient way to achieve a collection of molecules or an assembly of nanoparticles in solution [15,16]. We can easily achieve the above process only when the incident laser is on or off. In our previous work, we successfully obtained the assembly of gold nanoparticles on the LOF platform for SERS detection and developed a photothermal microreactor for copper ion detection, using thermophoresis based on easily modulated incident power. Our previous studies have demonstrated that the microfiber coated with photothermal nanomaterials can produce a sufficient temperature gradient and induce convection in the microfluidic chip. In this work, the synergistic effect of SERS and thermophoresis on a flexible, flat-based optical fiber to enhance the accuracy and reusability of Raman detection was discussed.

Ag or Au nanoparticles are widely used to prepare SERS-active substrates and produce rich “hot spots” for SERS enhancement arising from their remarkable surface plasmon properties [17,18,19,20,21]. Meanwhile, the Au or Ag nano/micro-island will induce thermophoresis in the solution based on the illumination of the launched laser light, beneficial for the transport of the analyte from another place to the “hotspot” in the solution. With the integration of the “hotspot” and the thermophoresis with the optical fiber, we can easily obtain a flexible SERS-based LOF sensor [22,23]. However, the strong oxidizing power of Ag [24] and the weak SERS activity of Au [25] limit the SERS enhancement based on the single noble metal substrate. The Ag/Au bimetallic system integrates the respective advantages and shows both significant Raman signal enhancement and good compatibility, attracting wide attention in the field of SERS [26,27,28,29]. 

Here, we construct a highly efficient and flexible SERS-based LOF sensor by integrating Ag/Au nanocap arrays obtained by Ag/Au coating polystyrene spheres (PS) on the optical fiber facet. The Ag/Au nanocap arrays show an obvious hierarchical structure and exhibit strong SERS activity, beneficial for analyte detection. We modified the morphologies of the Ag/Au nanocap and Ag film thickness to optimize the localized surface plasmon resonance (LSPR) effect. The results show that the sensor achieves low detection limits of 10^−7^ mol/L for 4-aminothiophenol (4−ATP) and methylene blue (MB), and 10^−9^ mol/L for Rhodamine 6G (R6G). 

## 2. Experimental Section

### 2.1. Materials

The 5 wt% aqueous suspensions of PS spheres with diameters of 120 nm were purchased from Hugebio Corporation, Shanghai, China. R6G, 4−ATP, MB, and ethanol were used without further purification.

### 2.2. Pretreatment of Optical Fiber

A multimode optical fiber with a core/cladding diameter of 62.5/125 µm was used to prepare a fiber-optic SERS sensor. Both ends of the fiber were cleaved using a fiber cutter to obtain flat facets; the fiber was then washed with deionized water and dried in an oven.

### 2.3. Preparation of Ag/Au Nanocap Arrays

PS nanospheres with a diameter of 120 nm were arranged on the fiber facet in a perfectly ordered hexagon using a gas–liquid interface self-assembly method. Monodisperse polystyrene colloidal particles (5 wt% aqueous suspension) were diluted 1:1 with ethanol, sonicated for 5 min, and then placed for use. Deionized water was carefully added dropwise to a sonicated hydrophilic glass slide. The colloidal suspension of PS spheres was dropped on one side of the glass slide so that the PS colloidal spheres could freely spread to the water surface on the hydrophilic glass slide. The deionized water under the PS sphere membrane was removed with filter paper. The PS colloidal spheres self-assembled into a hexagonal close-packed array due to the attractive capillary force together with the repulsive electrostatic force [30,31]. Then, the monolayer PS sphere film on the glass slide was transferred onto the surface of the deionized water in a clean beaker. The treated fiber substrate was immersed in the beaker with tweezers and placed directly under the monolayer PS spherical membrane. The fiber was lifted through the monolayer PS sphere membrane with tweezers so that the PS nanosphere array was transferred to the fiber facet. The 30 nm thick Au nanofilm was deposited on the top of the periodic PS nanospheres at a rate of 0.02 nm/s by vacuum thermal evaporation technology under a vacuum environment of 5 × 10^−4^ Pa. Due to the perfectly ordered hexagonal arrangement of PS nanospheres, the Au film was mainly deposited on the upper half of the PS nanosphere arrays to form an array of Au nanostructures.

Based on the above-mentioned fiber-optic probe with Au nanocap arrays as a platform, Ag/Au nanocap arrays were prepared on the facet of the optical fiber. The Ag nanofilm was deposited on the top of periodic Au nanostructures at a rate of 0.02 nm/s by vacuum thermal evaporation technology under a vacuum environment of 5 × 10^−4^ Pa. Thus, the preparation of fiber-optic SERS probes with Ag/Au nanocap arrays was completed.

### 2.4. Characterization 

The morphologies of the LOF SERS sensor were characterized with a field emission scanning electron microscope (FESEM, Sigma 500, Carl Zeiss, German). The Raman spectra of the analyte solution were acquired via a portable Raman spectrometer (Ava Spec-ULS-TEC, Avantes, The Netherlands). 

### 2.5. FDTD Calculations

The finite-difference time-domain (FDTD) method was used to simulate the electromagnetic field intensity of the LOF SERS sensor [15]. To simplify FDTD stimulation, three adjacent Ag/Au nanocaps were selected as the model. 

## 3. Results and Discussion

**Figure 1:**
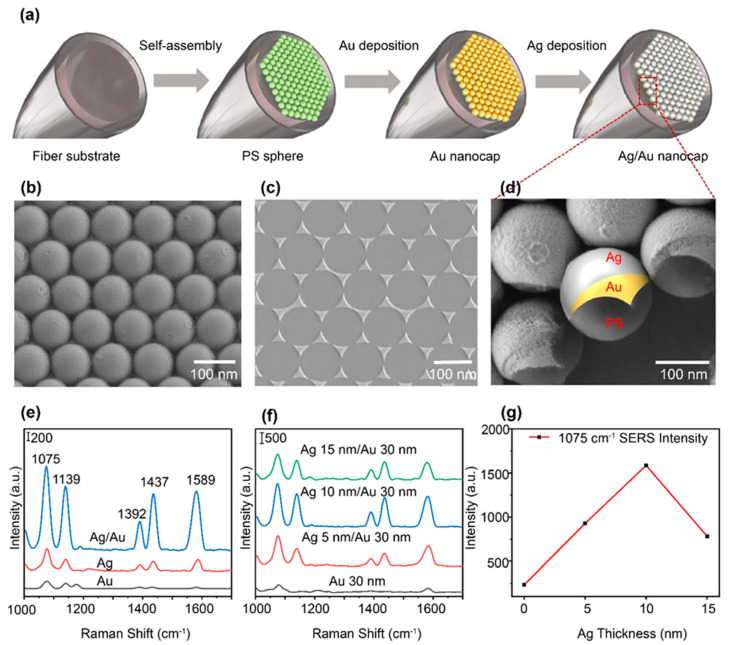
(**a**) Schematic diagram of the fabrication process of the LOF sensor. (**b**) SEM image of Ag/Au nanocap arrays on the fiber facet. (**c**) The SEM image of Ag/Au nanotriangles obtained by sonication of Ag/Au−coated nanospheres. (**d**) The highly enlarged SEM image of the Ag/Au nanocap bilayer on the PS sphere surface. (**e**) 4−ATP (10^−5^ M) Raman spectra obtained from LOF sensor with Ag/Au nanoarrays, Ag nanoarrays, and Au nanoarrays. (**f**) Raman spectra of 4−ATP (10^−5^ M) measured under different conditions of silver film thickness. (**g**) Corresponding dependence of Raman signal intensity on the thickness of silver.

To achieve the above device for SERS detection, we assembled NPs by using the clean fiber facet and deposited Ag/Au film on the surface to achieve both the photothermal effect and SERS. Figure 1a illustrates the SERS detection process on the fiber-optic probe based on the Ag/Au nanocap array (see the fiber Raman experimental setup in Figure S1 in the Supplementary Materials). For fiber Raman measurements, the fiber-optic probes were directly dipped in analyte solutions, and the SERS signals were excited by a laser with a wavelength of 785 nm at a power of 8 mW. Meanwhile, the SERS signals of the analyte solution were detected by a portable Raman spectrometer with an acquisition time of 2 s. This backscattering measurement method fully utilizes the advantages of fiber-optic SERS probes and realizes real-time, in situ, and remote measurement in the true sense. The SEM images in Figure 1b,d display the perfectly ordered hexagonal arrangement of PS spheres on the fiber facet, producing the possibility of uniform polygonal Ag/Au film on the fiber facet (Figure 1c). In order to obtain the image of the nanocap on the PS spheres, we immersed the fiber device in deionized water for ultrasonic cleaning for five minutes and obtained the image of an Ag/Au nanocap when the nearby nanosphere was removed. As shown in Figure 1d, a bilayer structure of the Ag/Au nanocaps can be clearly recognized from the high-magnification SEM images despite the complete overlap of Ag/Au film with polygonal Ag/Au film (Figure 1c), while the Ag/Au bilayer staggers to form the nanotriangles’ sharp corners which are beneficial for the photothermal effect and SERS [32]. The nanostructure of the bilayer therefore achieves the design goal of the device. 

In order to investigate the physical mechanism of SERS detection by fiber-optic probes, the finite-difference time-domain (FDTD) method was used to characterize the electric field intensity distribution of Ag/Au nanocap arrays on the optical fiber facet. We set the thickness of Au film as 30 nm, the thickness of Ag film as 5 nm, and the diameter of the PS microsphere as 120 nm. Under the excitation of a 785 nm laser, the “hot spots” distribution of the Ag/Au nanocap arrays on the optical fiber facet is shown in Figure 1b. We found significant field enhancement at the nanogap between adjacent Ag/Au nanocaps. In addition, temperature and liquid flow were numerically simulated based on the finite element method using COMSOL Multiphysics. As a 785 nm laser is coupled into the fiber at 8 mW, the metal film on the fiber surface leads to significant local heating on the near field based on the photothermal effect, creating a temperature gradient in the analytical solution (Figure 1c). Under the effect of temperature gradient, some heat convection effects formed in the solution, which directly made the solution move and also drove the particles in the solution to move. In this way, the aggregation and movement of analyte particles on the SERS hotspot on the fiber facet could be realized. The corresponding liquid velocity field near the fiber facet is shown in Figure 1d. The convergence and movement directions of analyte particles can be obtained from the simulation results.

The composition of the Ag film and Au film is an important factor for Raman signal enhancement. Au-monolayer, Ag-monolayer, and Ag/Au-bilayer films are fabricated on the fiber facet using nanosphere lithography. The test system is illustrated in Figure S1. The obtained Raman peaks appear at approximately 1075 cm^−1^, 1139 cm^−1^, 1392 cm^−1^, 1437 cm^−1^, and 1589 cm^−1^ for 4−ATP (10^−5^ M), which are consistent with previous reports [33,34] and reveal the reliability of the SERS sensor. Compared to both the Ag and Au monolayers, the bilayer film with 30 nm Au and 5 nm Ag exhibits a relatively high SERS signal about five times higher than that of the Ag monolayer and 10 times that of the Au monolayer (Figure 1e and Figure S2), revealing the high Raman enhancement of the bilayer film. The significant enhancement property of bilayer film is mainly attributed to the synergic effect of the plasmon on the interface of Ag/Au bilayer film.

Furthermore, the thicknesses of the Au and Ag films in the bilayer are other important factors for SERS enhancement. Employing 4−ATP (10^−5^ M) as a probe molecule, the enhanced Raman spectra of bilayer films with different Ag thicknesses (0 nm, 5 nm, 10 nm, and 15 nm) are presented in Figure 1f. It can be seen that the Raman signal intensity of the 4−ATP changes with the thickness of Ag film. For clarity, the variation in the intensity of the Raman peak at 1074 cm^−1^ 4−ATP solution with a concentration of 10^−5^ M is plotted in Figure 1g. We can see that the SERS peak of the 4−ATP molecule is significantly enhanced when depositing the Ag film on the surface of the Au nanocaps with a thickness smaller than 10 nm, and then it drops with a larger thickness of Ag film, revealing the optimum thickness of 10 nm of the Ag film in the bilayer nanocap nanostructure.

**Figure 2:**
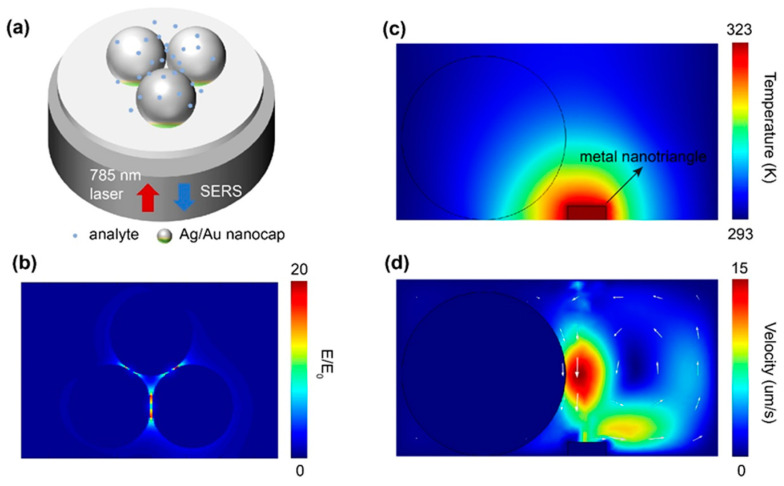
Physical mechanism of SERS-based LOF sensor. (**a**) Schematic diagram of the LOF sensor based on Ag/Au nanocap on optical fiber facet. (**b**) Simulated hotspot distributions based on the obtained Ag/Au nanocaps excited by laser light input from the fiber. Simulation of the thermal field distribution (**c**) and the liquid velocity field distribution (**d**) Flow of liquid near the metal film on the fiber facet.

The text continues here (Figure 2).

Figure 2 schematically presents the physical mechanism of Ag/Au nanogaps on the end face of the optical fiber. As shown in Figure 2a, the incident laser light will induce both photothermal effects at the surface of the Ag/Au film and SERS based on the nanogap between the Ag/Au film. The FDTD calculations confirm the above assumptions [35]. Under the excitation of a 785 nm laser, a rich “hot spots” distribution based on Ag/Au nanocap arrays on the optical fiber facet can be obtained at the nanogaps where the thickness of Ag/Au film is 30 nm/5 nm, and the diameter of the PS microsphere is 120 nm (Figure 2b). The significant field enhancement at the nanogap between adjacent Ag/Au nanocaps is beneficial for SERS. In addition, a 785 nm coupled laser light induces large local heating on both the fiber surface and nanocaps which are covered by Ag/Au film. Thanks to the thermophoresis effect, the temperature near the Ag/Au films dissipates gradually from 323 to 293 K (Figure 2c,d). The formed local temperature gradient imposes a nonuniform concentrate of the detected molecules that further drives the migration of the molecules in distant solution to the hot region, which is beneficial for the enhancement of the concentration at the ordered “hot spots” (Figure 2d).

**Figure 3:**
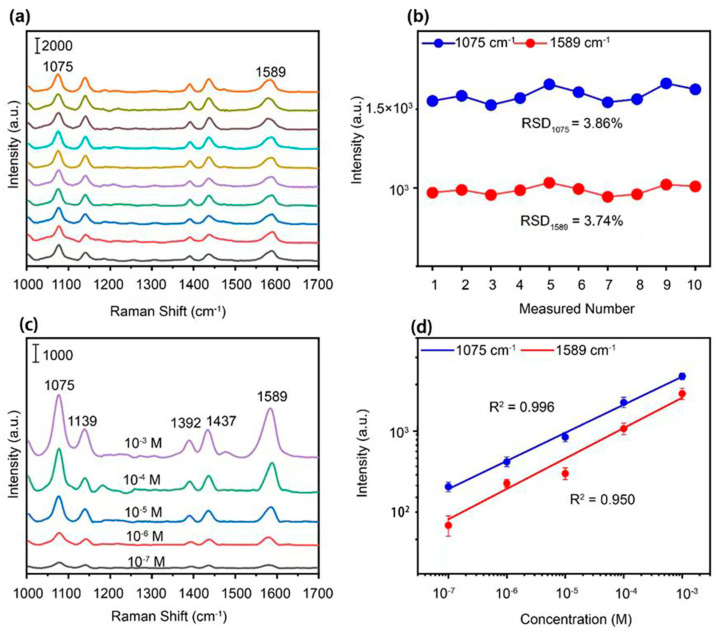
(**a**) Raman spectra of 4−ATP (10^−5^ M) solution measured by ten LOF sensors (in different colors). (**b**) SERS intensities obtained from (**a**) for peaks at 1075 cm^−1^ and 1589 cm^−1^. (**c**) Raman spectra of 4−ATP solutions with concentration of 10^−3^ M to 10^−7^ M, obtained from LOF sensors with Ag/Au nanocap arrays. (**d**) Log–log plots of SERS intensities versus concentrations at the peak locations of 1075 and 1589 cm^−1^.

The text continues here (Figure 3).

Such an achievement of the nanostructure allows us to characterize the basic SERS properties of this SERS-based LOF sensor. We first select the 4−ATP molecule as the target molecule to illustrate the LOF sensor performance. Figure 3a displays different SERS spectra based on sensors using the same 4−ATP solutions with concentrations of 10^−5^ M. The SERS intensities of two Raman peaks at 1075 cm^−1^ and 1589 cm^−1^ are plotted in Figure 3b to evaluate the stability of the devices based on the same preparation method. The relative standard deviation values for these two peak intensities are 3.86% and 3.74%, respectively, revealing high stability and reproducibility.

In order to assess the detection sensitivity of the optimized sensor, the SERS spectra of 4−ATP solutions were recorded at different concentrations from 10^−3^ M to 10^−7^ M, and are shown in Figure 3c. The results show that a visible SERS peak characteristic of 4−ATP can be observed at concentrations as low as 10^−7^ M, exhibiting an excellent SERS sensitivity. The good linear relationship between the peak intensity (lgI) of 4−ATP’s SERS spectra and the solution concentration (lgC) is presented in Figure 3d with a coefficient R^2^ = 0.996 at 1075 cm^−1^ and R^2^ = 0.950 at 1589 cm^−1^, further confirming the good stability of the sensor. Based on the experimental results, our strategy has been proven to be quantitative in in situ detection for a low concentration of targeted molecules in a short integration time of 2 s.

**Figure 4:**
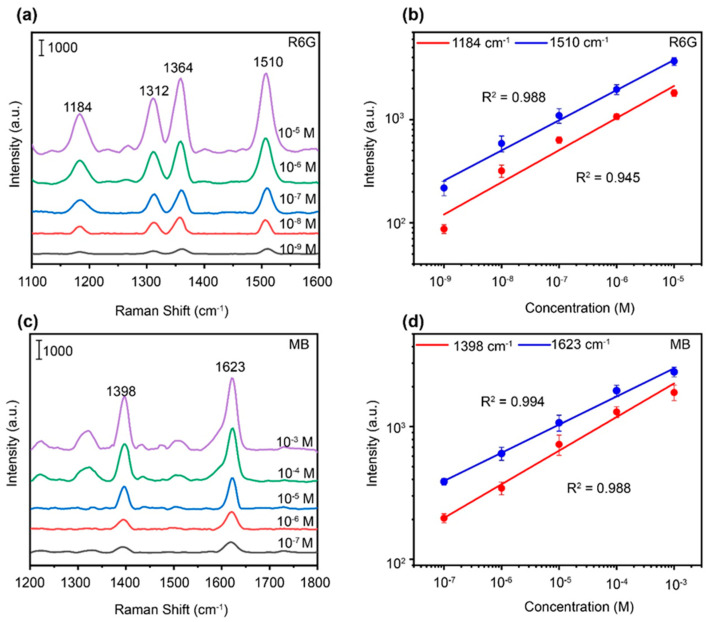
(**a**) Raman spectra of R6G solution at different concentrations. (**b**) Log−log plots of SERS intensities versus concentrations at 1184 and 1510 cm^−1^ for the R6G solution. (**c**) Raman spectra of R6G solution at different concentrations. (**d**) Log−log plots of SERS intensities versus concentrations at of 1398 and 1623 cm^−1^ for the MB solution.

For practical applications, it is necessary to study the universality of the SERS sensor in different molecular detections. We thus use this sensor for the detection of low-concentration rhodamine 6G (R6G) and methylene blue (MB) as the target molecules. The SERS-based LOF sensor shows sharp and distinct Raman peaks at low concentrations of 10^−9^ M for the R6G solution (Figure 4a,b) and 10^−7^ M for the MB solution (Figure 4c,d), revealing the good Raman enhancement of the sensor for probe molecules with low concentrations. The corresponding log–log plots of Raman peaks at 1184 and 1510 cm^−1^ for the R6G solution and at 1398 and 1623 cm^−1^ for the MB solution are further presented in Figure 4b,d, respectively. The good linear relationship between logarithm of intensity and concentration with the R^2^ larger than 0.94 confirms the stability of the sensor. Therefore, the SERS-based LOF sensor can be used for the quantitative detection of various probe molecules with good controllability and high sensitivity.

The average enhancement factor of the SERS-based LOF sensor can be calculated using the formula $EF=\left(I_{SERS}/N_{SERS}\right){\left(I_{RS}/N_{RS}\right)}^{-1}$, where $\;I_{SERS}$ and $I_{RS}\;$ represent the relative peak intensities of the SERS spectrum from the SERS-based LOF sensor and the Raman spectrum from a naked fiber probe, respectively. $N_{SERS}$ and $N_{RS}$ represent the corresponding numbers of molecules illuminated by the excitation laser in the above two environments [36]. We assume that the volume of analytes near the fiber facet contributes to the Raman signals. This formula can therefore be changed to $EF=\left(I_{SERS}/C_{SERS}\right){\left(I_{RS}/C_{RS}\right)}^{-1}$. We noted that in the measurements, the normal Raman signals of R6G are difficult to obtain with a bare fiber probe at low concentrations (e.g., 10^−6^ M), so we applied R6G with a high concentration of 10^−2^ M to obtain the Raman spectrum, as shown in Figure S3. The Raman peak intensity at 1510 cm^−1^ is selected to calculate the Raman enhancement factor, and the Raman enhancement factor is calculated to be 2 × 10^5^ for the optimized fiber-optic SERS probes with an ordered Ag/Au composite nanostructure. This is about equal to other optical manipulation methods based on Au film deposited glass [37] and plasmonic core-satellite nanostructures [38].

## 4. Conclusions

In conclusion, we have experimentally and theoretically demonstrated a SERS-based LOF sensor based on the Ag/Au nanogaps on the facet of an optical fiber with self-assembly PS spheres. The 10 nm Au film over 35 nm Ag film exhibits a higher Raman enhancement and produces a photothermal effect. Further experiments confirm the quantitative detection of pollutant solutions, such as 4−ATP, R6G and MB, with detection limit concentrations of 10^−7^, 10^−9^, and 10^−7^, respectively, and reveal good stability and reliable reproducibility. In the future, we need to combine this microfiber with microfluidic channels to further improve its utility, stability, and recycling efficiency.

## Data Availability

No data were used for the research described in the article.

## Supplementary Materials

The following supporting information can be downloaded at: https://www.mdpi.com/article/10.3390/polym15204107/s1, Figure S1: The schematic diagram of the setup in SERS detection; Figure S2: The dark Raman scattering noise generated by the fiber probe; Figure S3: Normal Raman spectrum of R6G (10^−2^ M) measured by using a naked fiber probe.
