# Supplementary material for: Lab-on-Fiber Sensors with Ag/Au Nanocap Arrays Based on the Two Deposits of Polystyrene Nanospheres
Source: Polymers (Basel). 2023 Oct 16;15(20):4107. doi: 10.3390/polym15204107 (PMC10610900; doi:10.3390/polym15204107)
Supplement: Supplementary file 1 [file polymers-15-04107-s001.zip › polymers-2644589-supplementary.pdf]

## Supporting Information

# Lab-on-fiber sensors with Ag/Au nanocap arrays based on the two deposits of Polystyrene nanospheres

Meng Shi [a, b, *], Shifang Gao [b], Liang Shang [b], Linan Ma [b], Wei Wang [a], Guangqiang Liu [b], Zongbao Li [c, d,*]

[a] School of Physical Science and Intelligent Engineering, Jining University, Qufu 273155, China

[b] Shandong Provincial Key Laboratory of Laser Polarization Technology, Qufu Normal University, Qufu 273165 Shandong, China

[c] Ministry of Education Key Laboratory of Textile Fiber Products, School of Materials Science and Engineering, Wuhan Textile University, Wuhan, 430200, China

[d] School of Materials and Chemical Engineering, Tongren University, Tongren 554300, China

* Corresponding Authors.

E-mail addresses: zongbaoli1982@163.com (Z. Li); philipyes@163.com (M. Shi)

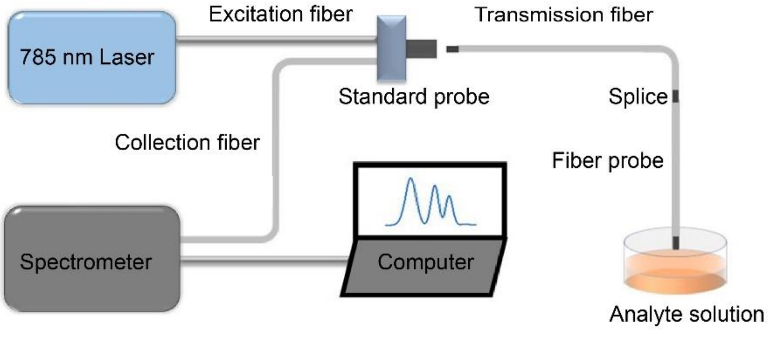


**Figure S1.** The schematic diagram of the setup in SERS detection.

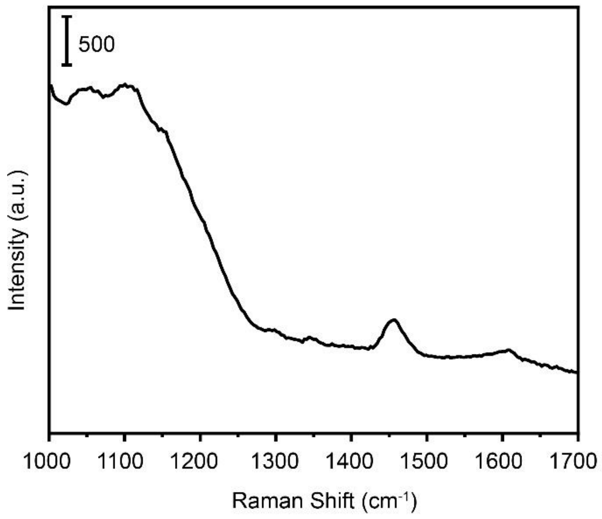


**Figure S2.** The dark Raman scattering noise generated by the fiber probe.

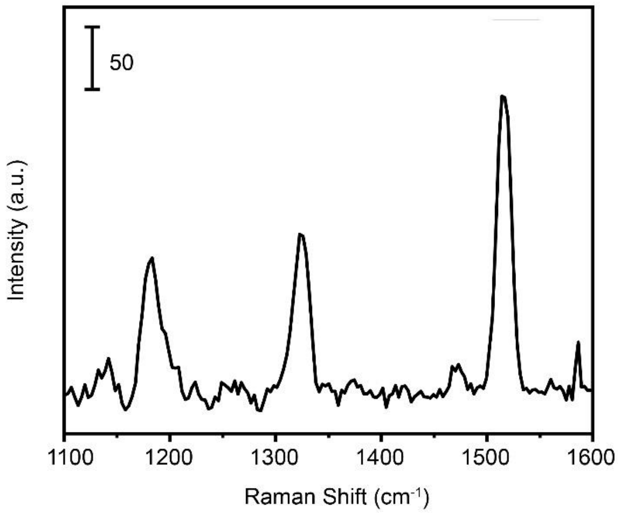


**Figure S3.** Normal Raman spectrum of R6G ($10^{-2}$ M) measured by using a naked fiber probe.
